# The Potential Link between Episodes of Diverticulitis or Hemorrhoidal Proctitis and Diets with Selected Plant Foods: A Case–Control Study

**DOI:** 10.3390/nu13061791

**Published:** 2021-05-24

**Authors:** Juan Flich-Carbonell, Antoni Alegre-Martinez, Jose L. Alfonso-Sanchez, Maria T. Torres-Sanchez, Segundo Gomez-Abril, Maria I. Martínez-Martínez, José M. Martin-Moreno

**Affiliations:** 1 Colorectal Surgery Section, General and Digestive Surgery Service, Dr. Peset University Hospital, 46017 Valencia, Spain; jflich@comv.es (J.F.-C.); mteresats@aecirujanos.es (M.T.T.-S.); sean99cartu@yahoo.com (S.G.-A.); 2 Department of Biomedical Sciences, Cardenal Herrera CEU University, Moncada, 46115 Valencia, Spain; antonimir2010@gmail.com; 3 Head of Preventive Medicine Service, University General Hospital of Valencia, 46014 Valencia, Spain; 4 Department of Preventive Medicine and Public Health, University of Valencia, 46010 Valencia, Spain; 5 Department of Nursing, Faculty of Nursing and Podiatry, University of Valencia, 46010 Valencia, Spain; M.Isabel.Martinez@uv.es; 6 INCLIVA and Clinical University Hospital, 46010 Valencia, Spain

**Keywords:** diverticulitis, hemorrhoids, vegetables, prevention, epidemiology, consumption

## Abstract

Diverticulitis and hemorrhoidal proctitis in the population are significant public health problems. We studied the potential association between the intake of certain plant foods and diverticulitis or hemorrhoidal episodes through a case–control study including 410 cases and 401 controls. We used a semiquantitative food frequency questionnaire. The intake was additionally quantified according to a 24 h recall. The plant foods or derived food products were categorized by their main chemical components into ethanol, caffeine/theine/theobromine, capsaicin, alliin, acids, eugenol, and miscellaneous foods such as curcumin. The mean score for overall intake of plant foods under consideration was 6.3 points, and this was significantly higher in cases (8.5) than in controls (4.1). Overall intake was similar in cases presenting with diverticulitis or hemorrhoidal proctitis. Cases had 13 times the odds of being in the upper quartile for overall intake (>7 points), compared to controls. Explanatory logistic regression models showed that the strongest association with diverticulitis and hemorrhoidal proctitis was shown by the chemical food group of capsaicin, followed by ethanol, eugenol, caffeine/theine/theobromine, and acids. Neither alliin nor miscellaneous food groups showed any association. High, frequent consumption of capsaicin, followed by ethanol, eugenol, caffeine/theine/theobromine, and acids increase the risk of diverticulitis and hemorrhoidal proctitis.

## 1. Introduction

Diverticulitis is characterized by inflammation of one or several adjacent diverticula and the surrounding colon. Patients with diverticulitis present with the acute or subacute onset of abdominal pain that is typically located in the left lower quadrant. If untreated, diverticular inflammation may resolve, become chronic, or progress, leading to bacterial translocation or even perforation of the colon wall at the inflamed site. The prevalence of diverticular disease increases with age, being reported as less than 10 percent in people younger than 40 years old and up to 66 percent in patients older than 80 years old. Diverticulitis, an inflammation or infection in a diverticulum can occur in 10–25 percent of these patients [1]. Moreover, it is the most usual complication of diverticular disease, affecting 10–25 percent of patients with diverticula. In the United States, the most common gastrointestinal tract diagnoses among hospitalized patients are diverticulitis and diverticular hemorrhage [2].

Hemorrhoids clinically are characterized by painless rectal bleeding during defecation with or without prolapsing anal tissue. It is defined as the symptomatic enlargement and distal displacement of the normal anal cushions. Hemorrhoids are therefore the pathological term to describe the abnormal downward displacement of the anal cushions causing venous dilatation. It is one of the most common anorectal diseases, can occur at any age and to any gender, and is reported to occur in more than half of the population over 50 years of age [3]. Hemorrhoids, also called piles, are masses or clumps of tissues that consist of muscle and elastic fibers with enlarged, bulging blood vessels and surrounding supporting tissues present in the anal canal of an individual. This condition is a common ailment among adults [4].

The high incidence of diverticulitis and hemorrhoidal proctitis episodes (this last one is 11% in the adult population) [5] in the population imposes important healthcare and economic burden. The etiology of these pathologies remains uncertain [6,7]. Hemorrhoidal disease refers to the state of symptoms attributed to the vascular cushions present in the anal canal, and this disease is closely related to acute diverticulitis that is an inflammation due to microperforation of a diverticulum. The diverticulum is a sac-like protrusion of the colon wall. Diverticulitis can present in about 10% to 25% of patients with Diverticulosis. Diverticulosis of the colon is a common disease in Western societies [7]. One of the curious theories is that posture during defecation is a risk factor for these diseases and would explain the increased prevalence in developed countries, as opposed to developing countries. In societies that have adopted Western toilets, people sit during defecation instead of squatting. The belief that squatting is a more natural position for defecation, and less likely to contribute to constipation and hemorrhoids, has led to the promotion of different toilet accessories. However, the pathogenesis of the process of these diseases is likely multifactorial, involving dietary habits, changes in colonic pressures and motility, and colon wall structural changes associated with aging.

The most widely accepted theory points to low fiber intake in the population as a causal factor [6,8], although other authors have proposed placing the blame on coffee and alcohol intake [9,10], smoking [11,12,13,14] pharmacological treatments [15,16], obesity and lack of physical activity [11,12,17], constipation, diarrhea, inflammatory bowel disease and irritable bowel syndrome [6,18,19,20,21], genetic predisposition [9,22], and geographic and ethnicity-related epidemiological factors [6,18,23]. However, the level of evidence is of very low certainty.

This study aims to assess whether the intake of certain plant foods or derived food products is a risk factor for symptoms of diverticulitis and hemorrhoidal proctitis.

## 2. Material and Methods

### 2.1. Study Design, Setting, and Population

We opted for an analytical observational case–control study, using a semiquantitative food frequency questionnaire (FFQ), focusing on the consumption of certain foods (frequency of intake and quantity consumed on intake days). The fieldwork was carried out in the Dr. Peset University Hospital, a public healthcare center with a catchment population of 380,000 people.

The institutional Clinical Research Ethics Committee approved the research project (80/18). All patients signed informed consent and participated freely as volunteers.

We used a case–control study since this design is well suited to the study question, comparing subjects who have the disease (the “cases”) with patients who do not have the disease but are otherwise comparable (the “controls”), in order to identify factors that may contribute to the disease or condition under study. Cases were defined as patients who attended the department of surgery with the following conditions: diagnosis of diverticulitis, hemorrhoidal proctitis, and or symptomatic hemorrhoids from June 2018 to September 2019. Controls were drawn from patients observed during the same period in the orthopedic surgery and traumatology, ophthalmology, and dermatology services (considering the patient history of colorectal conditions).

### 2.2. Interviews and Variables

Three investigators undertook one-on-one interviews with participants, using the same interview protocol. Variables included were age, gender, and intake patterns for seven groups of plant foods or derived food products, categorized by their chemical composition as follows:

Ethanol: spirits with high alcohol content and burning or harsh taste. Wine and beer were excluded;

Caffeine/theine/theobromine: drinks, soft drinks (e.g., colas or tea beverages), and foods (e.g., chocolate) with these bitter-tasting alkaloids. Decaffeinated products were excluded;

Capsaicin/piperine: plant foods with acids and alkaloids (varieties of peppers chili peppers) used to season foods (e.g., cold cut meats) and sauces with a spicy flavor;

Alliin: sulfoxide in garlic, onion, and other plant varieties with a spicy flavor;

Acids: foods with an acidic flavor, such as vinegar, pickled vegetables, orange, lemon, strawberry, cilantro, juice, soft drinks (with orange or lemon juices), and sauces that contain these;

Eugenol: clove, ginger, curry, cinnamon, and mustard, which contain acids and aldehydes with a sour, spicy, and bitter flavor;

Miscellaneous: curcumin, ginseng, cuminaldehyde (cumin), fenugreek or trigonelline (alhova), cinnamaldehyde (cinnamon), nutmeg acids and alkaloids, menthol, and other plant-based chemical products.

### 2.3. Measures and Scoring System

The scoring methodology used to quantify dietary intake patterns was designed as follows: “The food products or processed foods containing substances from different categories (sauces, soft drinks, sweets, and others) were counted a single time in the dominant group. All included plant food products consumed by the participants were recorded.”

The daily intake (24 h) was recorded for each plant food or derivative in one of two ways, according to patients’ responses: on a scale of 1–4 (no intake, low, moderate, or high intake) or as the number of units consumed (e.g., cups of tea/coffee/hot chocolate, ounces of chocolate, n soft drinks, n alcoholic drinks, n of oranges or lemons eaten or used to make juice, n of other food units). The frequency of intake was assessed as the number of days per week when the food product was consumed and scored as follows: 1 point, occasional or no consumption, or as the number of intake days per week (1–7). The value for 24 h intake was multiplied by the number of intake days per week to obtain a single score for each included food group.

To assign an overall score considering the intake patterns across the diverse range of included plant food products, we calculated a continuous variable by successively multiplying the intake score for each category and transformed the result into a log with the base of 10. For each participant, this composite score expressed the overall quantity and frequency of intake for all possible plant food risk factors included in the study.

### 2.4. Statistical Analysis

The mean score for overall intake of included plant food categories was 6.3 points (standard deviation [SD] 4.5). We categorized participants by quartiles, establishing four intervals: ≤3.00 points, 3.01 to 5.00 points, 5.01 to 7.00 points, ≥7.01 points.

Groups were compared by means of Pearson’s chi-squared test, Student’s t test, and Levene’s test, and predictive analysis with multiple logistic regression and odds ratios (ORs) with 95% confidence intervals (CIs). Quantitative data were expressed as mean (SD) and qualitative data as percentages. Before carrying out linear regression analyses, we assessed “normality” and checked the assumptions of a linear relationship and homoscedasticity.

Analyses were undertaken with IBM SPSS^®^, version 26.0 (IBM Corp. Released 2019. IBM SPSS Statistics for Windows, Version 26.0. IBM Corp: Armonk, NY, USA).

## 3. Results

A total of 872 patients (410 cases and 462 controls) were initially included and interviewed; however, 61 (13.2%) controls were excluded from the analysis because they had a history of gastroenterological pathologies under study. Thus, the final sample consisted of 811 patients: 410 cases and 401 controls (Table 1). The two groups were similar with regard to gender distribution and age, although the score for overall intake of the studied plant foods was higher among cases (case = 8.3 vs. 2.8 for controls).

Table 2 shows the distribution of pathologies among the cases. There were 24% more cases of hemorrhoids, compared to diverticulitis. In addition, among patients with hemorrhoids, 67 colonoscopies were performed, revealing 39 patients (15.6%) who also had diverticulosis. Similarly, 96 (59.6%) patients with diverticulitis had presented with episodes of acute hemorrhoids, and 17 of these were treated surgically. Participants with hemorrhoids were more frequently men and, on average, 6 years younger than the patients with diverticulitis, although these groups were similar in terms of the overall intake score for the included plant foods.

Mean intake scores for some individual plant foods and their most common derivatives were compared between cases and controls (Table 3), showing higher intake for most of these foods in cases. When the foods were categorized by chemical composition, there were significant differences between groups for all of them.

A comparison of the mean intake scores by quartiles (Table 4) shows that the progressively higher scores result in an increased OR, which reaches a value of 13.16 in the highest quartile. A predictive analysis using multiple regression (Table 5) was performed to assess the association between plant food categories and the presentation of symptoms. The final model indicated that capsaicin was the strongest predictor of the pathologies under study (OR 1.45), followed by ethanol (1.30), eugenol (OR 1.19), caffeine/theine/theobromine (OR 1.11), and acids (OR 1.08). The OR for age was near the null value. Neither alliin nor miscellaneous food groups showed any association.

## 4. Discussion

The FFQ is an advanced form of the checklist in the dietary history method and asks respondents how often and how much food they ate over a specific period [24]. This study was conceived to investigate dietary patterns in patients with diverticulitis and hemorrhoidal proctitis, specifically the intake patterns for certain plant foods and derived products (some of which have been studied by other authors [9,10]) with a sour, spicy, or bitter flavor. These products can be consumed directly or added to other foods such as meat, preserves, sauces, or processed foods and consumed in the form of sweets, tea, soft drinks, or other industrialized foodstuffs. The potential biological health benefits for some of these substances are under study [25,26,27,28]. However, a high concentration or quantity of certain chemical components can, in contact with conjunctiva [29,30] or mucous [31,32,33,34], provoke irritation, with varying degrees of secretion; connective edema; increased vascularization; and other effects such as cytotoxicity, necrosis, and ulceration of skin cells [35]. These effects support the hypothesis that concentrations of these chemical products in the residues of the colon and rectum may facilitate the pathophysiology [6,36] of the acute symptomatic period of diverticulitis and hemorrhoidal proctitis, which is characterized by edema in the connective tissue and vascular ecstasy. These processes lead to the appearance of symptoms (pain and/or hemorrhage) of varying intensity, persistence, and propensity for recurrence or complications such as ulceration, fissure, perforation, abscess, or fistula.

The intensely and harshly flavored plant foods included in the present study were more prominent in the diets of cases, compared to controls, both overall and in most cases at an individual level. Similarly, when the foods were grouped by chemical composition, patients with symptoms of diverticulitis and hemorrhoidal proctitis showed higher intake patterns. On considering the length of time over which the foods were consumed, we observed that few patients presented an acute symptomatic episode following several weeks of abundant, daily intake of the plant foods in question (for example, after vacationing in countries or regions where these foods formed an integral part of the local diet). Rather, the vast majority of cases reported moderate, regular intake of these products and few to no symptoms for months or years prior to the episode prompting the medical consultation. Age was included as a variable in the logistic regression model, showing that this was not a factor.

One limitation of the study was not physically examining participants in the control group to understand how many presented diverticulitis and hemorrhoids since these pathologies frequently manifest with few symptoms. However, we did exclude 13.2% of the control sample with a history of seeking medical care for such causes. We also observed that that subgroup presented similar overall intake scores to cases, and therefore, on exclusion from the comparative analysis, the OR increased for the quartiles with higher intake scores.

Another possible limitation is the research methodology, although it was based on that used by other studies that used standardized food frequency questionnaires with 24 h recall to identify differences in dietary patterns between populations and over time [37,38]. Taking into account the potential challenges of these studies [39,40], we developed a questionnaire adapted to our case–control design, extending the data collection period to 7 days and analyzing the data in the same way it was collected (without transforming them into grams or mg). We created a continuous variable using a numerical formula that considers the total intake of different plant foods according to the self-reported 24 h intake and its frequency (intake days/week) for each food category. Memory bias was controlled by conducting the retrospective survey for only 7 days prior. Possible biases in data collection, interviewer biases, and analysis would have been the same for both cases and controls; therefore, it is assumed that they did not produce important deviations.

Despite the difficulties and limitations mentioned and other possible reservations that may exist, our results suggest that people who rarely consume the plant foods studied are less likely to present symptoms of diverticulitis or hemorrhoidal proctitis. As intake of different products rises in quantity and frequency, so does people’s risk for these pathologies. This conclusion would explain why young patients with high intake patterns present symptoms, while older ones with low lifetime intake do not, showing no evidence of alterations in their colon, rectum, or anus on physical examination. Our results also suggest a shared pathophysiology and etiology between diverticulitis in the colon and hemorrhoids in the rectum and anus. The different alterations subsequently found in the mucosa and connective tissue of these organs depend on the anatomical differences in the vascular supply and distribution of the muscular layers on the gastrointestinal wall.

## 5. Conclusions

Abundant, frequent intake of certain plant foods and derived products appears to increase the risk of presenting symptoms of diverticulitis and hemorrhoids. Moderating the amount and frequency of intake in the population could help people decrease their risk for these symptoms.

Additional studies investigating these and other proposed risk factors and their interaction with the mucosa and connective vascular tissue of the colon, rectum, and anus could add to the evidence base supporting these observations.

## Figures and Tables

**Table 1:** Participant characteristics and overall intake of plant-based foods.

	Cases (N = 410)	Controls (N = 401)	Total (N = 811)	*p*-Value
Age in years, mean (SD)	55 (14)	55 (19)	55 (16)	0.98 *
Age groups, n (%)				0.05 ^†^
18–50 years	154 (37.6)	141 (35.2)	295 (36.4)	
51–65 years	153 (37.3)	129 (32.2)	282 (34.8)	
66–94 years	103 (25.1)	131 (32.7)	234 (28.9)	
Gender, n (%)				0.23 ^†^
Men	239 (58.3)	217 (54.1)	456 (56.2)	
Women	171 (41.7)	184 (45.9)	355 (43.8)	
Overall intake, mean (SD)	8.45 (5.33)	4.12 (1.64)	6.31 (4.51)	0.001 *

* t-test; ^†^ *χ*^2^.

**Table 2:** Demographic, clinical, and dietary characteristics of cases.

	Diverticulitis (N = 161)	Hemorrhoids (N = 249)	*p*-Value
Age, mean (SD)	59 (14)	53 (13)	0.001 *
Gender, n (%) Men Women	83 (51.6) 78 (48.4)	156 (62.7) 93 (37.3)	0.026 ^†^
Symptom severity, n (%) Simple Complicated ^‡^	115 (71.4) 46 (28.6)	183 (73.5) 66 (26.5)	0.64 ^†^
Overall intake, mean (SD)	8.98 (5.25)	8.10 (5.36)	0.10 *

* t-test; ^†^ *χ*^2^; ^‡^ perforation, abscess, fistula.

**Table 3:** Mean intake and frequency for individual food products and plant food categories by study group.

Plant Foods	Cases (N = 410)		Controls (N = 401)		2-Tailed *p*-Value *	Mean Difference	95% CI	
	N	Mean	N	Mean				
**Individual products**								
Chocolate	300	14.27	210	6.94	<0.001	7.33	5.64	9.02
Tea	82	11.92	53	6.25	<0.001	5.68	2.67	8.69
Lemon	133	11.63	65	7.95	<0.001	3.67	1.67	5.67
Soft drinks	82	12.52	50	8.92	0.045	3.60	0.08	7.12
Pepper	219	7.60	113	4.10	<0.001	3.50	2.34	4.65
Strawberry	44	6.98	8	3.75	0.034	3.23	0.26	6.20
Vinegar	223	9.37	170	6.32	<0.001	3.06	2.00	4.11
Cold cut meats ^†^	329	6.24	108	3.22	<0.001	3.02	2.09	3.96
Coffee	337	16.20	241	13.68	0.003	2.52	0.85	4.19
Pickled vegetables	301	8.48	211	6.44	<0.001	2.04	1.01	3.07
Raw onion	305	4.52	204	3.54	<0.001	0.98	0.60	1.36
Cooked onion	349	3.88	334	3.14	<0.001	0.74	0.50	0.98
Cooked garlic	296	3.72	295	3.05	<0.001	0.67	0.37	0.97
Curcumin	35	6.39	11	3.91	0.136	2.28	−0.75	5.30
Ginger	39	7.96	10	6.40	0.358	1.56	−1.82	4.95
Curry	41	4.48	10	3.00	0.135	1.48	−0.47	3.42
Orange	282	11.56	279	10.59	0.084	0.97	−0.13	2.08
Chili pepper	83	5.13	15	4.27	0.467	0.87	−1.49	3.22
Raw garlic	166	3.78	114	3.27	0.175	0.51	−0.23	1.25
Clove	30	4.28	0	—	—	—	—	—
***Plant foods grouped by chemical component***								
Ethanol	130	7.71	51	2.59	<0.001	5.12	3.11	7.13
Caffeine ^‡^	404	14.97	338	10.21	<0.001	4.76	3.61	5.90
Capsaicin	371	6.44	187	3.74	<0.001	2.70	2.05	3.36
Alliin	381	3.94	379	3.09	<0.001	0.84	0.11	0.63
Acids	391	10.09	363	8.13	<0.001	1.96	1.22	2.71
Eugenol	64	6.38	25	4.32	0.026	2.07	0.26	3.88
Miscellaneous	108	5.09	34	3.85	0.042	1.24	0.60	0.04

* Student’s t test; ^†^ seasoned with pepper and other spices in the capsaicin/piperine category; ^‡^ caffeine, theine, and theobromine. *Note*: The most frequently consumed individual foods are presented, excluding some with minimal use among both cases and controls (included under the chemical food group “Miscellaneous”).

**Table 4:** Comparison by quartiles of overall intake in cases versus controls.

Quartiles by Overall Intake Score, n (%)	Cases (N = 410)	Controls (N = 401)	Total (N = 811)	*p* *-Value	Odds Ratio (95% CI)
	n (%)	n (%)	n (%)		
Q1: 0.30–3.00	8 (2.0)	112 (27.9)	120 (14.8)	<0.001	0.05 (0.02 0.11)
Q2: 3.01–5.00	77 (18.8)	174 (43.4)	251 (30.9)	<0.001	0.30 (0.22 0.41)
Q3: 5.01–7.00	138 (33.7)	91 (22.7)	229 (28.2)	0.001	1.73 (1.23 2.36)
Q4: 7.01–27.7	187 (45.6)	24 (6.0)	211 (26.0)	<0.001	13.16 (8.33 20.83)

* *χ*^2^.

**Table 5:** Final model and interactions in predictive analysis of the influence of age and intake of plant food categories on the appearance of symptoms.

Predictor Variables: Case over Control	β Coefficient	β Standard Error	*p*-Value	OR	(95% CI)	
Age	0.014	0.006	0.018	1.01	1.00	1.03
Ethanol	0.263	0.062	<0.001	1.30	1.15	1.47
Caffeine *	0.101	0.018	<0.001	1.11	1.07	1.15
Capsaicin	0.370	0.067	<0.001	1.45	1.27	1.65
Alliin	0.099	0.115	0.39	1.10	0.88	1.38
Acids	0.075	0.038	0.048	1.08	1.00	1.16
Eugenol	0.172	0.058	0.003	1.19	1.06	1.33
Miscellaneous	0.019	0.068	0.78	1.02	0.89	1.16
Capsaicin-caffeine *	0.003	0.005	0.55	1.00	0.99	1.01
Acid-alliin	0.000	0.011	0.97	1.00	0.98	1.02

CI: confidence interval; OR: odds ratio; * caffeine, theine, and theobromine.

## Data Availability

Data available on request due to restrictions ethical.
